# Complex Dynamics of Osteoclast Formation and Death in Long-Term Cultures

**DOI:** 10.1371/journal.pone.0002104

**Published:** 2008-05-07

**Authors:** Timur Akchurin, Tayeb Aissiou, Naomi Kemeny, Erin Prosk, Nilima Nigam, Svetlana V. Komarova

**Affiliations:** 1 Faculty of Dentistry, McGill University, Montreal, Quebec, Canada; 2 Department of Mathematics and Statistics, McGill University, Montreal, Quebec, Canada; Center for Genomic Regulation, Spain

## Abstract

**Background:**

Osteoclasts, cells responsible for bone resorption, contribute to the development of degenerative, metabolic and neoplastic bone diseases, which are often characterized by persistent changes in bone microenvironment. We aimed to investigate the dynamics of osteoclast formation and death in cultures that considerably exceeded the length of standard protocol and to design a mathematical model describing osteoclastogenesis.

**Methodology/Principal Findings:**

RAW 264.7 monocytic cells fuse to form multinucleated osteoclasts upon treatment with pro-resorptive cytokine RANKL. We have found that in long-term experiments (15–26 days), the dynamics of changes in osteoclast numbers was remarkably complex and qualitatively variable in different experiments. Whereas 19 of 46 experiments exhibited single peak of osteoclast formation, in 27 experiments we observed development of successive waves of osteoclast formation and death. Periodic changes in osteoclast numbers were confirmed in long-term cultures of mouse bone marrow cells treated with M-CSF and RANKL. Because the dynamics of changes in osteoclast numbers was found to be largely independent of monocytes, a two-species model of ordinary differential equations describing the changes in osteoclasts and monocytes was ineffective in recapitulating the oscillations in osteoclast numbers. Following experimental observation that medium collected from mature osteoclasts inhibited osteoclastogenesis in fresh cultures, we introduced a third variable, factor *f*, to describe osteoclast-derived inhibitor. This model allowed us to simulate the oscillatory changes in osteoclasts, which were coupled to oscillatory changes in the factor *f*, whereas monocytes changed exponentially. Importantly, to achieve the experimentally observed oscillations with increasing amplitude, we also had to assume that osteoclast presence stimulates osteoclast formation.

**Conclusions/Significance:**

This study identifies the critical role for osteoclast autocrine regulation in controlling long-term dynamic of osteoclast formation and death and describes the complementary roles for negative and positive feedback mediators in determining the sharp dynamics of activation and inactivation of osteoclasts.

## Introduction

Osteoclasts are cells responsible for bone destruction in metabolic, degenerative and neoplastic bone disorders. Therapeutic compounds aimed at blocking osteoclast formation and resorptive activity, are widely used for treatment of these diseases. However, the need in developing new therapeutics is still great as well as diverse in its goals. First, more potent osteoclast blockers are needed to treat irreversible severe destruction of mineralized tissues in rheumatoid arthritis, periodontitis and metastatic bone disease [1], [2]. Second, for treatment of osteoporosis, the compounds that would “normalize” rather than totally block osteoclast activity are desired [2], [3]. Moreover, bone related side effects of numerous medications, including corticosteroids, immunosuppressants and anticonvulsants [4] provide ongoing need to assess potential effects of new therapies on bone cells, including osteoclasts. To assist in drug development and testing, different types of in vitro osteoclast formation and activity assays are commonly used for screening pharmacological compounds [5], [6].

Osteoclasts are terminally differentiated multinucleated cells of hematopoetic origin, formed by fusion of osteoclast precursors of monocyte-macrophage lineage. Cytokines RANKL (receptor activator of nuclear factor κB ligand) and M-CSF (macrophage colony-stimulating factor) have been identified as necessary and sufficient to induce osteoclastogenesis. Murine monocytic cell line, RAW 264.7, is widely used to study osteoclastogenesis [6]–[8], since these cells require only stimulation with RANKL to form multinucleated cells, whereas bone marrow or peripheral monocytes need to be treated with a combination of M-CSF and RANKL to achieve osteoclastogenesis. Upon stimulation with RANKL, monocytes differentiate and fuse forming giant multinucleated osteoclast-like cells, the process that requires 4–5 days to accomplish. Osteoclasts can be observed in cultures for 2–4 days, after which they die primarily by apoptosis [9], [10]. The efficiency of osteoclastogenesis in vitro is rather variable, however it rarely reaches 100%, therefore undifferentiated mononuclear cells can be readily detected together with mature osteoclasts. In contrast to terminally differentiated osteoclasts, monocytes are capable of proliferating in the culture, and they die primarily by apoptosis. Both monocyte proliferation and death have been previously reported to be affected by RANKL [11]. Better understanding of the mechanisms governing the dynamics of changes in osteoclast and monocyte numbers will improve predictive power of in vitro assays and will provide new information regarding the regulation of bone resorption in vivo.

Mathematical methods are now well acknowledged as integral part of biomedical research, where they are used in data analysis, predictive modeling and simulation modeling. One particular aspect of simulation modeling is the potential to create models that may be employed to perform experiments in silico, thus providing cost- and animal-saving means for assessing the impact of potential stimulators and inhibitors on the biological process of interest. In this regard, a mathematical model accurately describing the process of osteoclast formation is potentially of significant utility.

We [12], [13], and others [14], [15] have previously developed mathematical models of bone turnover, which describe the dynamics of changes in populations of different bone cells at the site undergoing bone remodeling. Although useful in their power to explain and predict different general phenomena, these models are far removed from routine experimental conditions, resulting in uncertainty in parameter estimations, and are of little use in simulating specific in vitro experiments.

The goal of this study was to design a mathematical model describing temporal changes in monocyte and osteoclast numbers, and to estimate model parameters based on direct correlations with the experiments. One of the important questions in the mathematical modeling is concerned with the steady state of the system, whereas the majority of experimental data capture only initial dynamics of the process. In order to obtain more detailed data on the long-term dynamics of monocytes and osteoclasts, we performed experiments that considerably exceeded the length of standard osteoclast cultures. Unexpectedly, we found that osteoclast numbers change in a manner much more complex than can be predicted by current knowledge. In particular, in a large proportion of experiments we observed synchronized waves of osteoclast formation and death. To account for such behavior, we introduced feedback controls in our model, and demonstrated that negative feedback is critical for obtaining oscillatory behavior in the system, whereas positive feedback is required to achieve experimentally observed osteoclast oscillations with increasing amplitude.

## Results

### Long term dynamics in monocyte-osteoclast cultures

**Figure 1:**
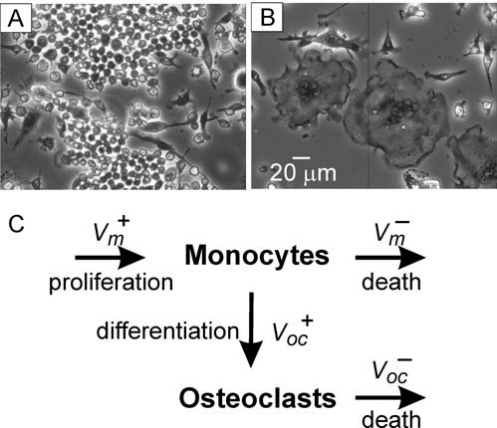
Osteoclasts are formed by fusion of monocytic precursors. A) Phase contrast micrograph of undifferentiated RAW 264.7 cells grown for 5 days without additions. B) Multinucleated osteoclast-like cells in the culture of RAW 264.7 cells treated for 5 days with RANKL (50 ng/ml). C) Schematic presentation of the processes occurring during osteoclast formation in vitro. Monocytes are depicted to undergo proliferation with the rate V_m_^+^ and cell death with the rate of V_m_^−^. Osteoclasts are formed by fusion of monocytes with the rate of V_oc_^+^. Osteoclast death occur with the rate V_oc_^−^.

**Figure 2:**
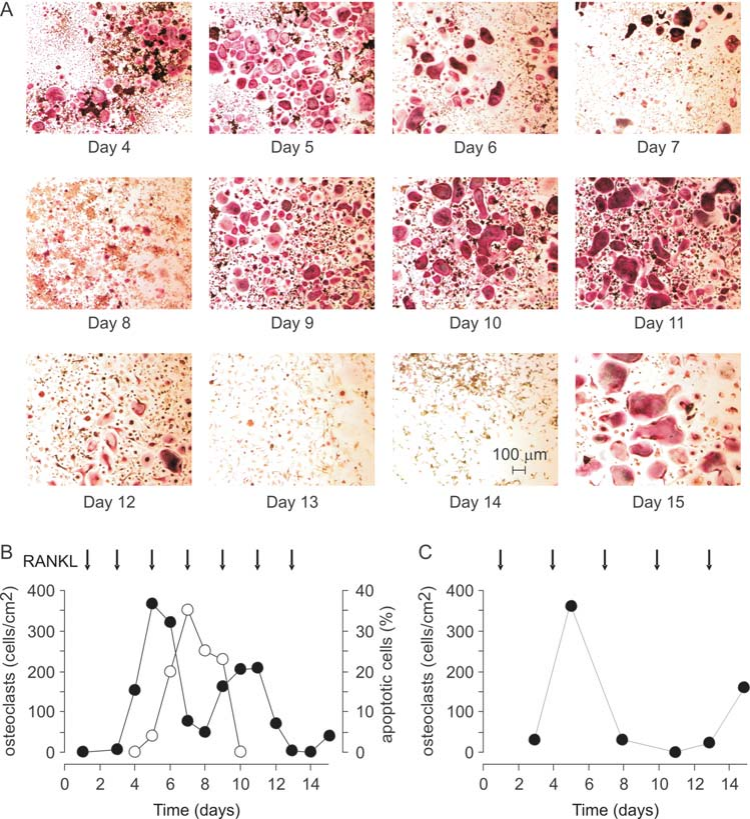
Synchronized waves of osteoclast formation and death observed in long-term cultures. A, B) RAW 264.7 cells were treated with RANKL 50 ng/ml during 15 days. Samples were fixed, and either stained for TRAP to assess the numbers of TRAP-positive multinucleated osteoclasts, or stained with DAPI to assess nuclear morphology. A) Micrographs of TRAP-stained samples taken at indicated days. Calibration bar applies to all images. B) *Black circles:* changes in osteoclast numbers during 15 days of culture; *open circles:* percentage of osteoclasts exhibiting nuclear fragmentation (apoptotic cells), normalized to the total number of osteoclasts. C) Mouse bone marrow cells were treated with MCSF (20 mg/ml) and RANKL (50 ng/ml) during 15 days. Samples were fixed, stained for TRAP, and numbers of TRAP-positive multinucleated osteoclasts were counted. Arrow indicates days when RANKL was added.

To assess long-term dynamics in osteoclast cultures, we treated RAW 264.7 with RANKL (Fig. 1) for 15–26 days, which is significantly longer than the standard protocol of 5–7 day osteoclast culture. We observed that treatment with RANKL leads to formation of multinucleated osteoclast-like cells that stain positive for an osteoclast marker, tartrate-resistant acid phosphatase (Fig. 2A). First osteoclasts appeared in culture on day 4–5 after plating. Osteoclast numbers remained high for 2–3 days and then started to decline. The decrease in osteoclast numbers was accompanied by the appearance of multinucleated cells with distorted morphology, absent nuclei, and unclear cell periphery, indicating osteoclast death. Osteoclast death, likely by apoptosis, was confirmed by an increase in the percentage of osteoclasts exhibiting nuclear fragmentation (Fig 2B). To our surprise, when we cultured cells for a longer time, we often observed a second wave of osteoclast formation, when after the temporary decline osteoclast numbers increased again (Fig 2). In several experiments, a third wave of osteoclast formation was also evident. Since RAW 264.7 cells are an immortalized cell line, we have also isolated bone marrow monocytic cells and characterized the dynamics of changes in osteoclast numbers in long-term primary cultures treated with RANKL and MCSF. We have found that similarly to RAW 264.7 cells, persistent stimulation of primary bone marrow monocytes with MCSF and RANKL leads to periodic changes in the numbers of osteoclasts (Fig 2C). It is of interest to note that in many experiments with RAW 264.7 cells, and especially in primary cultures, we have noticed that the size of osteoclasts tend to increase in subsequent waves of osteoclastogenesis, compared to the first wave.

### Characterization of long-term dynamics in osteoclast cultures

We next varied initial monocyte plating density and concentration of RANKL in long-term cultures of RAW 264.7 cells. We found that the long-term dynamics of changes in osteoclast numbers differed from experiment to experiment. Whereas in some experiments only single peak of osteoclast formation was observed, in other experiments clear oscillatory changes in osteoclast numbers were evident. To analyze the patterns of osteoclast dynamics, we pooled together 46 experiments that lasted from 15 to 26 days and were performed with different plating densities or different RANKL treatment. Since the amplitude of osteoclast formation was quite variable, for each single experiment we normalized the osteoclast numbers at different times by a maximum observed in that experiment, and limited the time duration to 17 days since this was the time frame for the majority of experiments.

**Figure 3:**
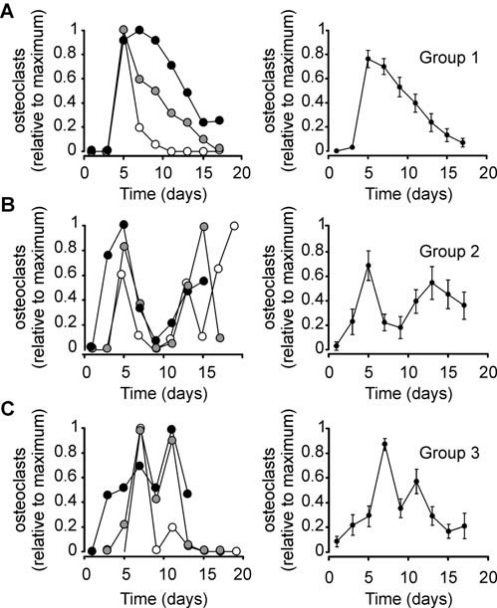
Complex dynamics of osteoclast formation and death observed in osteoclast cultures. RAW 264.7 cells were treated with RANKL, the samples were fixed at different days and the numbers of TRAP-positive multinucleated osteoclasts were assessed. 49 single experiments were normalized to the maximum number of osteoclasts observed in each experiment and binned for a two-day sampling interval. All experiments were divided into 3 groups. A) Group 1 included experiments that exhibited only one peak of osteoclast formation. Left – examples of 3 of 19 individual experiments belonging to group 1. Right – average changes in normalized osteoclast count with time; data are mean±SEM, n = 19 single experiments from 8 different plating dates. B) Group 2 included experiments that exhibited 2 peaks divided by at least 2 points, which had osteoclast count of less then 20% of either peak. Left – examples of 3 of 14 individual experiments belonging to group 2. The experiments were aligned for the time of the first maximum, which in different experiments occurred on day 3 (white circles), day 5 (gray circles) and day 7 (black circles). Right – average changes in normalized osteoclast count with time; data are mean±SEM, n = 14 single experiments from 9 different plating dates, all 14 experiments were aligned for the time of the first maximum. C) Group 3 included experiments that exhibited 2 peaks divided by just one point which had osteoclast count of less then 20% of either peak. Left – examples of 3 of 13 individual experiments belonging to group 3. The experiments were aligned for the time of the first maximum, which in different experiments occurred on day 5 (white circles), day 7 (gray circles) and day 11 (black circles). Right – average changes in normalized osteoclast count with time; data are mean±SEM, n = 13 single experiments from 9 different plating dates, all 13 experiments were aligned for the time of the first maximum.

We next divided 46 single experiments into 3 groups depending on the dynamics observed in each experiment. In group 1, we combined 19 experiments that exhibited only one peak of osteoclast formation (Fig. 3A). In group 2, we combined 14 experiments that exhibited 2 peaks divided by at least 2 points, which had an osteoclast count of less than 20% of either peak (Fig. 3B). In group 3, we combined 13 experiments that exhibited 2 peaks divided by just one point, which had an osteoclast count of less than 20% of either peak (Fig. 3C). In groups 2 and 3 the peaks in different experiments often did not coincide in time, resulting in significant smoothing when average osteoclast count in these groups was assessed. However, when we aligned the time of the first maximum in all the experiments in groups 2 and 3, we have found that the average osteoclast count captures the oscillatory changes observed in individual experiments (Fig 3B, C right), suggesting that in contrast to the initial dynamics of osteoclast formation, which may depend on specific experimental conditions, later dynamics of osteoclast changes are likely governed by the same intrinsic mechanism. For further analysis we combined experiments in group 2 and 3 as a single oscillating group. From 27 experiments in oscillating group, in 10 the amplitude of the second peak was less than 50% of the first peak, in 9 experiments the amplitude of the second peak was more than 50% but less than 150% of the first peak, and in 8 experiments the amplitude of the second peak was more than 150% of the first peak.

### The effect of initial conditions and RANKL treatment on long-term osteoclast dynamics

**Figure 4:**
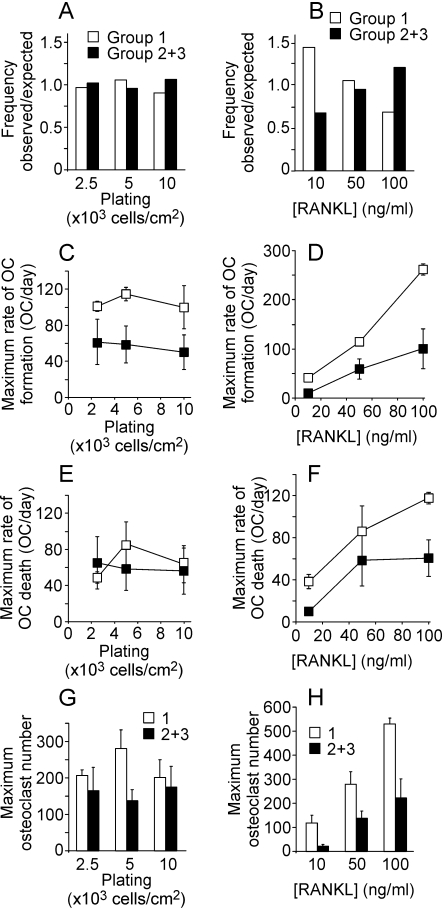
Comparison of non-oscillating and oscillating groups. The experiments were divided into non-oscillating group, which contained 19 experiments and oscillating group, which contained 27 experiments (combined groups 2 and 3). Within each group, experiments were divided according to the experimental conditions (plating density and RANKL treatment), and the following parameters were assessed: A, B) We compared the ratio of the proportion of experiments performed with specific conditions in non-oscillating (white bars, Group 1) and oscillating (black bars, Group 2+3) groups to the proportion of experiments performed with that condition in all experiments. A) Plating densities did not affect long-term dynamics of osteoclast cultures. B) RANKL concentration significantly affected the probability of experiment to belong to oscillating group. P<0.05, assessed by χ^2^ goodness of fit test. C, D) Maximal rate of osteoclast formation was estimated in each experiment and plotted as a function of plating density (C) or RANKL concentration (D). E, F) Maximal rate of osteoclast death was estimated in each experiment and plotted as a function of plating density (E) or RANKL concentration (F). G, H) Maximal number of osteoclast formed in each experiment and plotted as a function of plating density (G) or RANKL concentration (H). C–H) Data are mean±SEM, number of independent experiments are: RANKL (R) 50 ng/ml, plating density (p.d.) 5×10^3^ cells/cm^2^: n = 7 (group 1), n = 9 (group 2+3); R 50 ng/ml, p. d. 2.5×10^3^ cells/cm^2^: n = 4 (group 1), n = 6 (group 2+3); R 50 ng/ml, p. d. 10×10^3^ cells/cm^2^: n = 3 (group 1), n = 5 (group 2+3); R 10 ng/ml, p. d. 5×10^3^ cells/cm^2^: n = 3 (group 1), n = 2 (group 2+3); R 100 ng/ml, p. d. 5×10^3^ cells/cm^2^: n = 2 (group 1), n = 5 (group 2+3).

We next assessed the effects of the plating density and RANKL treatment on the long-term osteoclast dynamics in non-oscillating and oscillating groups. We first examined if plating density or RANKL concentration affects the probability of the development of oscillations in osteoclast numbers (Fig 4A, B). Using χ^2^ goodness of fit test, we compared the observed frequency of appearance of each experimental condition in oscillating or non-oscillating groups to the frequency of each condition in the whole experimental series. We have found that when we considered the experiments performed under different plating densities, the proportion of experiments performed with specific conditions in non-oscillating and oscillating groups was similar to the proportion of experiments performed with these conditions in all experiments (Fig 4A). However, when we considered variation in RANKL treatment, we have found that higher proportion of experiments performed with low concentration of RANKL (10 ng/ml) was in a non-oscillating group. Respectively, experiments performed with high concentration of RANKL (100 ng/ml) developed oscillatory behavior more frequently. Thus, RANKL concentration significantly affected the probability of the experiment to exhibit oscillations in osteoclast number.

We next investigated if plating density or RANKL treatment affects the rates of osteoclast formation and death and if these effects are different between oscillating and non-oscillating groups. In each experiment we calculated the maximal rate of osteoclast formation, the maximal rate of osteoclast death and the maximal number of osteoclasts formed over the duration of each experiment. We have found that whereas plating density did not affect these parameters (Fig 4C, E, G), increase in RANKL concentration led to a strong increase in the maximal rate of osteoclast formation (Fig 4D), and maximal number of osteoclasts formed (Fig 4H), as well as, to a smaller degree, to an increase in the maximal rate of osteoclast death (Fig 4F). Interestingly, in all experimental conditions, maximal rate of osteoclast formation in the oscillating group was found to be reduced compared to the non-oscillating group

### Model development and testing

#### Model 1

We have shown that in long-term osteoclast cultures different types of dynamics can be detected. Whereas in some experiments only a single peak of osteoclast formation was observed, in other experiments several waves of osteoclast formation and death were evident. Moreover, all oscillating experiments were divided approximately evenly between three groups: 37% exhibited a decrease in amplitude of the second wave, 33% exhibited similar amplitude of both waves and 30% exhibited an increase in the amplitude of the second wave. We observed that increase in RANKL stimulation was associated with an increase in the probability of development of oscillatory dynamics. Based on our experimental findings, we aimed at developing a model which would capture oscillatory changes in osteoclast numbers and would suggest which parameter (or combination of parameters) is likely to be associated with the appearance of oscillations and may be involved in determining the extent of damping.

First we constructed the model based on Figure 1:
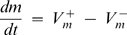
(1)

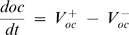
(2)where *m* and *oc* represent the numbers of monocytes and osteoclasts at the time *t* respectively; 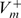 and 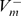 are the rates of monocyte formation and removal, and 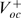 and 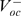 are the rates of osteoclast formation and removal. We further described the rates of monocyte and osteoclast formation and removal using linear dependences in the following general form:
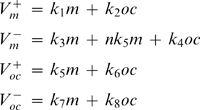
(3)where *k*_1_ *m* is the rate of monocyte proliferation, proportional to the current number of monocytes, *k*_1_>0; *k*_2_ *oc* is a potential effect of osteoclasts on monocyte formation; *k*_3_ *m* is the rate of monocyte death, *k*_3_>0; *k*_4_ *oc* is a potential effect of osteoclasts on monocyte removal; *k*_5_ *m* is the rate of osteoclast formation from monocytes, *n* is used to account for fusion, which takes more than one monocyte to form one osteoclast, *k*_5_>0; *k*_6_ *oc* is a potential effect of osteoclasts on osteoclast formation; *k*_7_ *m* is a potential effect of monocytes on osteoclast death; and *k*_8_ *oc* is the rate of osteoclast death, *k*_8_>0.

**Figure 5:**
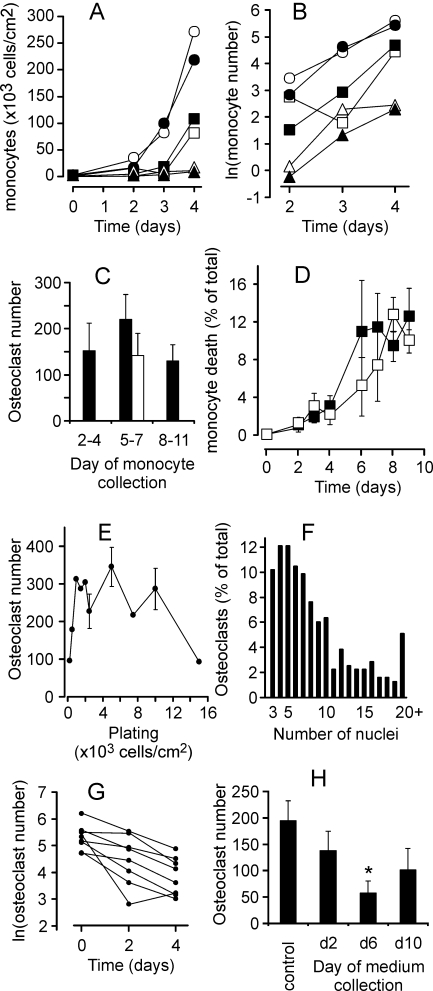
Estimation of parameters characterizing the dynamics of monocytes and osteoclasts. RAW 264.7 cells were plated at the density of 2.5×10^3^ cells/cm^2^ (triangles), 5×10^3^ cells/cm^2^ (squares), 10×10^3^ cells/cm^2^ (circles) and cultured either untreated (open symbols) or in the presence of RANKL (50 ng/ml, closed symbols). A–D) At indicated times the monocytes were collected from parallel samples and numbers of live and dead cells were counted. A) Changes in monocyte number with time are similar in RANKL treated and untreated cultures. B) Linear dependence of ln(monocyte number) on time indicates first order exponential dynamics for monocyte proliferation. C) The monocytes collected at indicated days from parallel samples, were re-plated on new wells at a density of 5×10^3^ cells/cm^2^ and treated with RANKL (black bars) or cultured without RANKL (open bar) for additional 5 days, when the samples were fixed and the numbers of TRAP-positive multinucleated osteoclasts were assessed. Data are mean±SEM, n = 5 independent experiments. D) Numbers of trypan blue positive (dead) monocytes were assessed at each time point and presented as a percentage of total number of monocytes. Data are mean±SD, n = 3 replicates. E) RAW 264.7 cells were plated at the indicated density and cultured in the presence of RANKL (50 ng/ml) for 5 days, when the samples were fixed and the numbers of TRAP-positive multinucleated osteoclasts were assessed. Data are means of 3 replicates for all densities except 2.5×10^3^, 5×10^3^, and 10×10^3^ cells/cm^2^, when data are mean±SEM, n = 9 independent experiments. F) In 3 independent experiments the number of nuclei per osteoclast was assessed in ∼100 osteoclasts per experiment. The data are percentage of osteoclast containing certain number of nuclei from the total of 315 osteoclasts. G) The rate constant of osteoclast death was estimated form the linear dependence of ln(osteoclast number) on time, with day 0 representing the day when maximum of osteoclasts was formed in each experiment. H) During 3 independent experiments, the medium was collected at the indicated day in the end of two-day culture period. RAW 264.7 cells were plated at the density of 5×10^3^ cells/cm^2^ and treated with RANKL (50 ng/ml) either without further addition (control) or supplemented with 10% osteoclast conditioned medium collected on indicated day. On day 5 the samples were fixed and the numbers of TRAP-positive multinucleated osteoclasts were assessed. Data are mean±SEM, n = 4 independent experiments, p<0.05, assessed by student t-test.

To estimate *k*_1_ and *k*_2_, we plated monocytes at different cell densities, and either treated them with RANKL, or cultured untreated for 4 days, while taking a daily sample to estimate the monocyte number in the cultures (Fig. 5A). In these experiments monocyte numbers reached a plateau at the density of ∼5×10^5^ cells/cm^2^, likely representing the capacity of the dish, when at higher densities some cells lost attachment and were unintentionally removed while changing media. We have found that either in the absence or presence of RANKL (and consequently osteoclasts, which appear on day 3–4), the monocyte numbers change exponentially, with a similar time constant. Thus, we estimated *k*_1_ as a slope of the ln*m*(t) dependence (Fig. 5B) as *k*_1_≅1.2 *day* ^−1^, and *k*_2_ as negligible, *k*_2_ = 0. We also investigated if the responsiveness of monocytes to RANKL may change during osteoclast culture; however we have found that the monocytic cells taken at different time from cultures containing osteoclasts are able to form osteoclasts with similar efficiency (Fig. 5C).

To assess *k*_3_ and *k*_4_, we estimated the percentage of dead monocytes as a proportion of total number of monocytes in untreated cultures or cultures treated with RANKL (Fig. 5D). We have found that the rate of monocyte death plateaued at ∼10% of total number of monocytes. We observed some trend for temporary increase in monocyte death in the presence of RANKL, although the difference was within experimental error. We estimated *k*_3_≅0.1 *day* ^−1^. We could not immediately estimate or discard *k*_4_ on the basis of experimental data, only evaluated it as likely positive.

We next estimated *k*_5_, *n* and *k*_6_. Based on the data we obtained in experiments with varied monocyte plating densities, the rate of osteoclast formation does not depend on the number of monocytes in the range of monocyte plating densities of 2.5–10×10^3^ cells/cm^2^ (Fig. 4C). When we have varied the plating densities from 250 to 15000 cells/cm^2^, we have found that the dependence is bell-shaped: at very low plating densities the number of osteoclasts formed are directly proportional to the plated monocyte number, whereas very high monocyte concentration becomes inhibitory for osteoclast formation (Fig. 5E). These data are in good agreement with the dependence of the rate of osteoclast formation on the number of monocytes reported previously [17]. We described the rate of osteoclast formation in the following form: 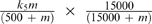, which in the range of *m*<500 is approximated as 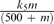, at the range of 500<*m*<15000 is approximated as *k*_5_, and in the range of *m*>15000 is approximated as 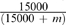. We estimated *k*_5_ to vary from 20 to 500 cells/day depending on RANKL treatment. The factor *n* relates to the monocyte fusion, which results in the removal of several monocytes from the monocyte population to add one osteoclast to the osteoclast population. We assessed the distribution of osteoclasts according to the number of nuclei contained by each (Fig. 5F). In total, 315 osteoclasts from 3 independent experiments contained approximately 2660 nuclei, resulting in estimated *n* = 8, as an average number of monocytes taken for formation of one osteoclast. The experimental data did not allow us neither for immediate exclusion of *k*_6_, nor for it estimation, which we left undefined to assess if it would have an influence on system dynamics. We considered the process that the component *k*_6_ *oc*, representing a potential effect of osteoclasts on osteoclast formation, may describe. It is generally acknowledged that osteoclasts are terminally differentiated cells that cannot proliferate. The alternative process of splitting the 6-nucleated osteoclast to form two 3-nucleated osteoclasts has never been described (in contrast to an opposite – forming bigger osteoclast from two multinucleated small osteoclasts). Thus, in the process of osteoclastogenesis this component likely represents the effect of the presence of osteoclasts on osteoclast formation from monocytes. As such, it would result in an osteoclast-dependent decrease in monocyte number taken to form the osteoclasts, which is represented by the component *k*_4_ *oc* in the equation describing the changes in monocyte number. Therefore, we assumed that the components *k*_4_ *oc*and *k*_6_ *oc* describe the same process, where *k*_4_ = *nk*_6_,

To estimate *k*_7_ and *k*_8_, we assessed the slope of the function ln*oc*(t), where the numbers of osteoclasts were taken starting at the first peak (Fig. 5G), and estimated *k*_8_ = 0.3 *day* ^−1^. No difference were observed in the rate of osteoclast death in cultures differed in monocyte densities, suggesting that k_7_ is negligible, *k*_7_ = 0.

Taking into account the parameter estimates, we arrived at the following model:
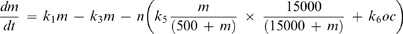
(4)

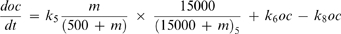
(5)


**Figure 6:**
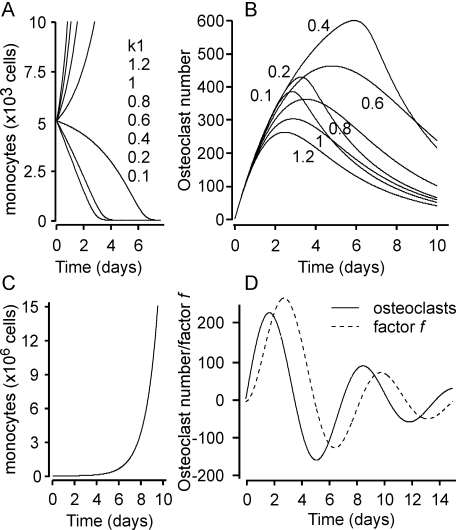
Dynamics of changes in monocyte and osteoclast numbers predicted by the mathematical model. A, B) Simulation of changes in monocyte number (A) and osteoclast number (B) obtained using the two-species model described by the equations (4–5), with the following parameters: *k*_1_ = 0.2; 0.4; 0.6; 0.8; 1; 1.2; *k*_3_ = 0.1; *k*_5_ = 300; *k*_6_ = 0; *k*_8_ = 0.3; *n* = 8. C, D) Simulation of changes in monocyte number (C) and osteoclast number (D) obtained using the three-species model described by the equations (7–9), with the following parameters: *k*_1_ = 1; *k*_3_ = 0.1; *k*_5_ = 300; *k*_6_ = 0.5; *k*_8_ = 0.3; *k*_9_ = 1; *k*_10_ = 1; *k*_11_ = 0.5; *n* = 8

Simulation of osteoclast and monocyte dynamics demonstrates that monocyte numbers either exponentially increase, when the rate of monocyte proliferation is higher than the rate of osteoclast formation, or decrease to 0, when the rate of osteoclast formation is higher then the rate of monocyte proliferation (Fig. 6A). In contrast, in both situations osteoclasts change in a similar way, first increasing in numbers and later decreasing to 0 either due to inhibition by high number of monocytes, or due to lack of monocytes to produce osteoclasts (Fig. 6B). In the range of monocyte values 500<*m*<1500, the system is approximated by the linear model:
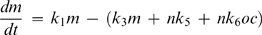
(6)

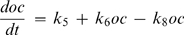
(7)or presented in a matrix form:
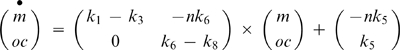
(8)The eigenvalues of this matrix are λ_1_ = *k*_1_–*k*_3_ and λ_2_ = *k*_6_–*k*_8_, real values at any value of *k_i_*, demonstrating that no oscillations can be achieved in this model.

#### Model 2

**Figure 7:**
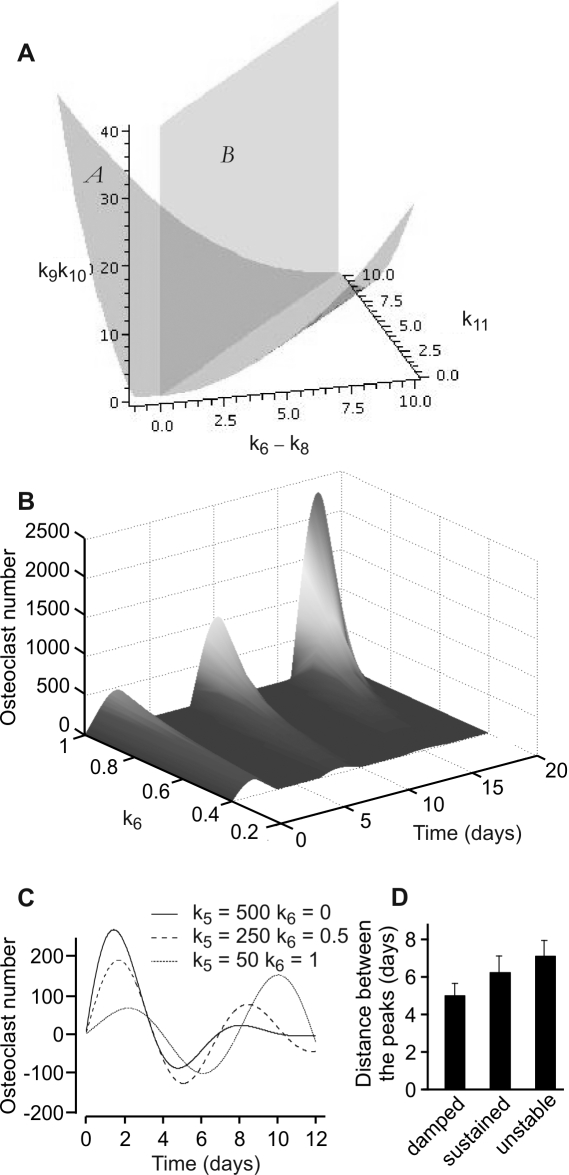
Positive feedback on osteoclast formation is critical for obtaining oscillations with increasing amplitude. A) Parametric portrait of the system in the space of parameters *k*_9_ *k*_10_; *k*_6_–*k*_8_; *k*_11_. Bifurcation surface *(A)*, described by equation (*k*_6_–*k*_8_–*k*_11_)^2^−4*k*_9_ *k*_10_ = 0, separates the regions of non-oscillatory (below the surface), and oscillatory (above the surface) behavior. Bifurcation surface *(B)*, described by equation *k*_6_–*k*_8_–*k*_11_ = 0, separates the regions of exponential growing and decaying behavior for osteoclasts and factor *f*. B) Increase in the value of parameter *k*_6_ results in development of oscillations of osteoclast numbers with increasing amplitude. C) Simultaneously increasing the value of parameter *k*_6_ and decreasing the value of parameter *k*_5_ allows simulation of experimental observation that the first peak is generally lower in experiments where osteoclasts oscillate with increasing amplitude compared to experiments without oscillations or with damped oscillations of osteoclasts. D) The 27 experiments where oscillations were observed were classified as damped oscillations, if the amplitude of the second peak was less then 50% of the first peak (n = 10), sustained oscillations, if the amplitude of the second peak was more than 50% but less then 150% of the first peak (n = 9), or unstable oscillations if the amplitude of the second peak was more then 150% of the first peak (n = 8), and the distance between 2 maximums was identified. Data are mean±SEM.

To assess the possibility that a factor produced during osteoclast culture may have an effect on osteoclast formation, we collected the medium at different times of osteoclast culture, then added this medium (10% of fresh medium) to the freshly seeded monocytes and induced osteoclastogenesis with RANKL (50 ng/ml). We have found that medium collected at day 6, when many mature osteoclasts are present had significant inhibitory effect on osteoclast formation (Fig. 5H). Thus, we introduced a third variable to the model, which describes the action of a negative regulator of osteoclastogenesis, factor *f* that is produced by osteoclasts, and removed proportionally to its value. The dynamics of the system are now described by the following model:
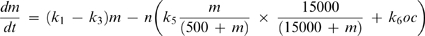
(9)

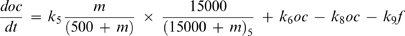
(10)

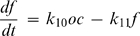
(11)In the range of monocyte values 500<*m*<15000 the model can be approximated by the linear model:
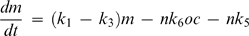
(12)

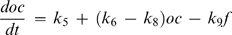
(13)

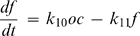
(14)or presented in a matrix form:
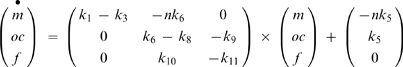
(15)The eigenvalues of this matrix are
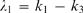
(16)

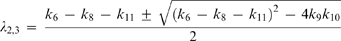
(17)where λ_1_ characterizes the exponential dynamics of monocytes, and λ_2,3_ characterize the coupled dynamics of osteoclasts and factor *f*. The development of oscillations in the model is determined by the determinant of equation (17), with oscillation present when (*k*_6_–*k*_8_–*k*_11_)^2^−4*k*_9_ *k*_10_<0 (Fig. 6C, D). The 3-dimensional parametric portrait of the system demonstrating the plane separating the regions of non-oscillatory and oscillatory behavior in the space of parameters *k*_6_–*k*_8_, *k*_11_ and *k*_9_ *k*_10_ is presented on the Fig. 7A. When (*k*_6_–*k*_8_–*k*_11_)^2^−4*k*_9_ *k*_10_<0, the type of the oscillations – damped, sustained or with increasing amplitude – is determined by the sign of *k*_6_–*k*_8_–*k*_11_. The oscillations are damped if *k*_6_–*k*_8_–*k*_11_<0, and develop with increasing amplitude if *k*_6_–*k*_8_–*k*_11_>0. In this model, parameter *k*_6_, representing a positive effect of osteoclasts on the rate of osteoclast formation is essential in order to obtain oscillations with increasing amplitude (Fig. 7B), since both *k*_8_ and *k*_11_ are positive rate constants of removal of osteoclasts and factor *f* respectively. It is of interest to note that while an increase in *k*_6_ led to development of the oscillations with increasing amplitude, it also resulted in the increase in amplitude of the first peak. In contrast, in our experiments we had observed that when oscillations with increasing amplitude developed, the first peak was generally lower than in experiments without oscillations or with damped oscillations. To simulate these experiments, we had to simultaneously increase *k*_6_ and decrease *k*_5_ (Fig. 7C), suggesting that *k*_6_ and *k*_5_ are not independent parameters, but likely part of the same circuit designed to obtain robust osteoclastogenesis. Another interesting feature of these simulations is that the period of oscillations increases when the type of oscillations changes from damped, to sustained, to unstable. We reviewed our experimental data and confirmed that a similar trend is observed experimentally (Fig 7D).

## Discussion

In this study we endeavored to develop a mathematical model describing the process of osteoclastogenesis in vitro. We have found that in the system containing only two cell types, monocytes and osteoclasts that are formed by fusion from monocytes, the dynamics of changes in osteoclast numbers in long-term (15–26 days) cultures were remarkably complex and qualitatively variable in different experiments. The spectrum of observed behavior was consistent with the change in the nature of the steady state of the system in different experiments, between stable nodes, and stable and unstable foci. Since long-term dynamics in osteoclast cultures were found to be largely independent of monocytes, a two-species linear ODE model describing the changes in osteoclasts and monocytes was ineffective in recapitulating the oscillatory behavior of osteoclast numbers. We observed that medium collected from mature osteoclasts exhibited an inhibitory effect on the osteoclastogenesis in fresh cultures; therefore we introduced a third variable, describing the changes in osteoclast-derived factor *f*, which has inhibitory effects on osteoclasts. This model allowed us to simulate the oscillatory behavior in osteoclasts, which was coupled to oscillatory changes in the factor *f*, whereas monocytes changed monotonically in an exponential manner. In addition, the positive effect of osteoclasts on their formation was critical in order to recapitulate the experimentally observed oscillations with increasing amplitude. Thus, we have found that two critical assumptions are needed in order to reproduce experimental behavior in our model: 1) there is a factor that is produced by osteoclasts and has an inhibitory effect on osteoclast formation and 2) osteoclast presence has a positive effect on osteoclast formation.

An important question to ask is what could be the nature of these regulatory feedbacks. RANKL and OPG, produced by osteoblasts, are widely believed to be the most important regulators of osteoclastogenesis [18], [19]. While not in any way diminishing the importance of the RANKL pathway, our study demonstrates that the long-term dynamics of osteoclast changes predominantly depend on autocrine signaling by osteoclasts. In this regard, the studies specifically aimed at uncovering autocrine regulators of osteoclastogenesis [20], [21] have found several positive and negative regulators of osteoclast formation. Among the positive regulators were interleukin 6 [21], [22], calcium-dependent phospholipid-binding protein annexin-II [20], [21], [23], and Adam8, transmembrane disintegrin and metalloproteinase implicated in cell-cell interactions by acting through integrin α9β1 [24], [25]. In addition, TGFβ, produced by osteoclasts as well as liberated from bone during resorption, has been shown to directly stimulate osteoclast formation at low concentrations [26]. Negative autocrine regulators of osteoclast formation include interferon β, which is induced in osteoclast precursors by RANKL and was shown to suppress excessive osteoclastogenesis [27], [28], nitric oxide, also induced by RANKL [29], as well as osteoclast inhibitory peptides I and II [21], [24], [30]. Our study suggests that both positive and negative autocrine feedbacks are concurrently involved in regulation of osteoclastogenesis. Whereas negative feedback is carried out by the soluble factors produced by mature osteoclasts, the positive feedback is of more complex nature, likely representing the ability of mature osteoclast to stimulate differentiation and fusion of osteoclast precursors by direct cell-cell interaction. Membrane bound factors, such as annexin-II, and ADAM-8, fit the profile of the positive osteoclast regulator suggested by the mathematical model. Another important suggestion of the model is that positive feedback becomes evident only when osteoclastogenesis is sub-optimal, suggesting that experimentally it will be observed only in situations when RANKL stimulation induces insufficient response from osteoclast precursors. Thus, during osteoclast differentiation, the positive feedback assures the robust increase in osteoclastogenesis upon stimulation, whereas negative feedback limits the effect of the stimulus, together resulting in sharp dynamics of activation and inactivation of osteoclasts.

There are several physiological and pathological situations, where periodic activation of osteoclasts has been detected. The most prominent example is Paget's disease of bone, which is characterized by periodic local osteolysis followed each time by bone formation by osteoblasts [31]. Interestingly, each subsequent cycle is characterized by higher extent of bone resorption and bone formation, thus resembling the oscillations with increasing amplitude. The underlying pathology of Paget's disease of bone is believed to be associated with defect in the cells of osteoclast lineage [31]. Another example is osteoclast recruitment during physiological tooth eruption. During the eruption of rat first mandibular molar, a first wave of osteoclast formation occurs at day 3 postnatally [32], [33]. Interestingly, a second wave of smaller amplitude occurs at day 10 [33], [34], and finally tooth erupts on day 18. Whereas a first wave of osteoclastogenesis was shown to depend on factors produced by the dental follicle, such as MCSF and RANKL, the stimulation underlying the second wave is currently unresolved. Notably, the timing between two waves of osteoclast formation during tooth eruption is similar to that observed in our experiments, suggesting that potential stimulus for the second wave of osteoclast formation may be intrinsic to osteoclasts, rather than dependent on external factors. It is also known that intracellular calcium oscillations play important roles in mediating osteoclast responses to RANKL [35], and in osteoclast movement and spreading [36]. However the time scale of oscillation of intracellular calcium or calcium-dependent currents is in the order of seconds to minutes, whereas oscillations in osteoclast numbers occur with the periodicity in the order of days, suggesting that the association between the two phenomena is unlikely.

Osteoclasts are cells of hematopoetic origin. It is of interest to mention that several hematopoetic disorders associated with oscillations in cell numbers are known. Chronic eosinophilic leukemia results in oscillations in white blood cells, platelets, and bone marrow cellularity with a period of 60 days [37]. Cyclic thrombocytopenia is a rare syndrome characterized by oscillations in the blood platelet numbers with a period of 28–57 day, sometimes associated with oscillations of other hematopoetic cells, such as reticulocytes and neutrophils [38], [39]. Cyclic neutropenia is characterized by oscillations in circulating neutrophil numbers which are often accompanied by oscillations in the platelets and reticulocytes [40], [41]. Such disorders appear to be inherent for the hematopoetic cells, which are characterized by the ability to rapidly respond to demands by changing their numbers, and are lacking cell-cell contact and therefore relying on potent soluble mediators to provide positive and negative feedback regulation. This study identifies osteoclasts as rightful members of this cell lineage and suggests that autocrine factors regulating osteoclast formation are critical for bone physiology and pathology.

Overall, our study provides new information about the process of osteoclastogenesis by taking into account long-term dynamics of osteoclast changes. It constitutes the first step towards the development of a mathematical model suitable for *in silico* experimentation and highlights the difficulties in creating such models in a biologically accurate manner. In times of wide prevalence of large scale modeling [42], our study provides a word of caution for developing and interpretation of these models and calls for better understanding of dynamic regulation of elementary processes.

## Materials and Methods

### RAW 264.7 cells

The RAW 264.7 mouse monocytic cells (American Type Culture Collection) were cultured in DMEM with 1.5 g/L sodium bicarbonate, 4.5 g/L glucose, with glutamine (Wisent Inc. Cat No 319-020-CL), supplemented with 1% sodium pyruvate (Wisent Inc. Cat No 600-110-EL), 1% antibiotics (10,000 IU penicillin, 10,000 µg/ml streptomycin, Wisent Inc. Cat No 450-201-EL) and 10% fetal bovine serum (Hyclone, SH 30396-03). For osteoclast formation, RAW 264.7 cells were plated on coverslips at a density of 2.5×10^3^, 5×10^3^, or 10×10^3^ cells/cm^2^ (as indicated), and 24 h later (day 1) supplemented with RANKL (10, 50 or 100 ng/ml, as indicated). Cultures were maintained for 15–26 days, supplemented with fresh medium every other day. Samples (1–3 coverslips per time point) were taken either every day or every 2 days and fixed in 4% paraformaldehyde. Osteoclasts were identified as multinucleated (more than 3 nuclei) cells that stained positive for tartrate-resistant acid phosphatase (TRAP) (Sigma, Cat No 387A). To examine monocytes, osteoclast cultures were treated with CellStripper (Mediatech Inc. Cat No 25-056-CI) for 5–10 min, the cell suspension was gently mixed and collected into eppendorf tubes and the monocyte number was counted using hemocytometer, with dead cells identified using the trypan blue exclusion test. For re-plating, monocytes were centrifuged, resuspended in fresh medium, plated at the density 5×10^3^ cells/cm^2^ and treated with RANKL (50 ng/ml) for 5 days.

### Bone marrow cultures

To assess osteoclast formation in primary cultures, mouse bone marrow cells were isolated from the long bones of six weeks old C57BL/6J mice as described previously [16]. The procedures were approved by the McGill University Animal Care Committee according to guidelines established by the Canadian Council on Animal Care. Mouse bone marrow cells were collected from the femur and tibia, seeded on glass coverslips at 5×10^3^ cells/cm^2^ and cultured in DMEM supplemented with 1% sodium pyruvate, 1% L-glutamine, 10% fetal bovine serum, and 1% antibiotics (10,000 IU penicillin, 10,000 µg/ml streptomycin). Mouse bone marrow cultures were treated with MCSF (20 mg/ml) and RANKL (50 ng/ml) for 15–44 days, and supplemented with fresh medium every other day. The samples were taken every 2 days, fixed in 4% paraformaldehyde and osteoclasts were identified as multinucleated (more than 3 nuclei), TRAP-positive cells.

### Osteoclast death

As a measure of cell death, nuclear fragmentation was examined in osteoclasts differentiated from RAW 264.7 cells using the nuclear stain 4′,6-diamidino-2-phenylindole (DAPI, Invitrogen). Fixed cells were washed in phosphate buffered saline (PBS), permeablilized with 0.1% Triton –X100 for 10 min, washed in PBS, stained for 30 min for F-actin with Bodipy 558/568 phalloidin (Invitrogen) to visualize cell border, washed in PBS and counterstained for 5 min with DAPI. 10–15 random images per time point were acquired using inverted fluorescence microscope (Nikon) and the Volocity software (Improvision®, UK). For each time point the total number of osteoclasts and the number of osteoclasts exhibiting nuclear fragmentation were assessed.

### Statistical Analyses

Independent experiments were defined as experiments performed at different plating dates, thus starting from different passages of Raw 264.7 cells. Single experiments were performed with particular conditions, such as specific plating density and specific RANKL treatment. Within most independent experiments we varied either plating density or RANKL concentration; therefore several single experiments belonged to one independent experiment. Data are presented as traces of single experiments or as a mean±standard error of the mean, with sample size (n) indicating the number of independent experiments. Differences were assessed by t-test or by χ^2^ goodness of fit test, and accepted as statistically significant at P<0.05.

## References

[1] Stern PH (2007). Antiresorptive agents and osteoclast apoptosis.. J Cell Biochem.

[2] Grey A (2007). Emerging pharmacologic therapies for osteoporosis.. Expert Opin Emerg Drugs.

[3] Karsdal MA, Martin TJ, Bollerslev J, Christiansen C, Henriksen K (2007). Are nonresorbing osteoclasts sources of bone anabolic activity?. J Bone Miner Res.

[4] Lawson J (2002). Drug-induced metabolic bone disorders.. Semin Musculoskelet Radiol.

[5] Takahashi N, Udagawa N, Kobayashi Y, Suda T (2007). Generation of osteoclasts in vitro, and assay of osteoclast activity.. Methods Mol Med.

[6] Collin-Osdoby P, Yu X, Zheng H, Osdoby P (2003). RANKL-mediated osteoclast formation from murine RAW 264.7 cells.. Methods Mol Med.

[7] Goya M, Ishii G, Miyamoto S, Hasebe T, Nagai K (2006). Prostate-specific antigen induces apoptosis of osteoclast precursors: potential role in osteoblastic bone metastases of prostate cancer.. Prostate.

[8] O'Sullivan S, Naot D, Callon K, Porteous F, Horne A (2007). Imatinib promotes osteoblast differentiation by inhibiting PDGFR signaling and inhibits osteoclastogenesis by both direct and stromal cell-dependent mechanisms.. J Bone Miner Res.

[9] Pereverzev A, Komarova SV, Korcok J, Armstrong S, Tremblay GB (2007). Extracellular acidification enhances osteoclast survival through an NFAT-independent, protein kinase C-dependent pathway.. Bone.

[10] Yun JH, Kim CS, Cho KS, Chai JK, Kim CK (2007). (-)-Epigallocatechin gallate induces apoptosis, via caspase activation, in osteoclasts differentiated from RAW 264.7 cells.. J Periodontal Res.

[11] Bharti AC, Takada Y, Shishodia S, Aggarwal BB (2004). Evidence that receptor activator of nuclear factor (NF)-kappaB ligand can suppress cell proliferation and induce apoptosis through activation of a NF-kappaB-independent and TRAF6-dependent mechanism.. J Biol Chem.

[12] Komarova SV, Smith RJ, Dixon SJ, Sims SM, Wahl LM (2003). Mathematical model predicts a critical role for osteoclast autocrine regulation in the control of bone remodeling.. Bone.

[13] Komarova SV (2005). Mathematical model of paracrine interactions between osteoclasts and osteoblasts predicts anabolic action of parathyroid hormone on bone.. Endocrinology.

[14] Kroll MH (2000). Parathyroid hormone temporal effects on bone formation and resorption.. Bull Math Biol.

[15] Lemaire V, Tobin FL, Greller LD, Cho CR, Suva LJ (2004). Modeling the interactions between osteoblast and osteoclast activities in bone remodeling.. J Theor Biol.

[16] Wani MR, Fuller K, Kim NS, Choi Y, Chambers T (1999). Prostaglandin E2 cooperates with TRANCE in osteoclast induction from hemopoietic precursors: synergistic activation of differentiation, cell spreading, and fusion.. Endocrinology.

[17] Ishida N, Hayashi K, Hoshijima M, Ogawa T, Koga S (2002). Large scale gene expression analysis of osteoclastogenesis in vitro and elucidation of NFAT2 as a key regulator.. J Biol Chem.

[18] Boyce BF, Xing L (2007). Biology of RANK, RANKL, and osteoprotegerin.. Arthritis Res Ther.

[19] Wada T, Nakashima T, Hiroshi N, Penninger JM (2006). RANKL-RANK signaling in osteoclastogenesis and bone disease.. Trends Mol Med.

[20] Takahashi S, Reddy SV, Chirgwin JM, Devlin R, Haipek C (1994). Cloning and identification of annexin II as an autocrine/paracrine factor that increases osteoclast formation and bone resorption.. J Biol Chem.

[21] Roodman GD (2006). Regulation of osteoclast differentiation.. Ann N Y Acad Sci.

[22] Reddy SV, Takahashi S, Dallas M, Williams RE, Neckers L (1994). Interleukin-6 antisense deoxyoligonucleotides inhibit bone resorption by giant cells from human giant cell tumors of bone.. J Bone Miner Res.

[23] Menaa C, Devlin RD, Reddy SV, Gazitt Y, Choi SJ (1999). Annexin II increases osteoclast formation by stimulating the proliferation of osteoclast precursors in human marrow cultures.. J Clin Invest.

[24] Choi SJ, Han JH, Roodman GD (2001). ADAM8: a novel osteoclast stimulating factor.. J Bone Miner Res.

[25] Rao H, Lu G, Kajiya H, Garcia-Palacios V, Kurihara N (2006). Alpha9beta1: a novel osteoclast integrin that regulates osteoclast formation and function.. J Bone Miner Res.

[26] Quinn JM, Itoh K, Udagawa N, Hausler K, Yasuda H (2001). Transforming growth factor beta affects osteoclast differentiation via direct and indirect actions.. J Bone Miner Res.

[27] Takayanagi H (2007). Osteoimmunology: shared mechanisms and crosstalk between the immune and bone systems.. Nat Rev Immunol.

[28] Takayanagi H, Ogasawara K, Hida S, Chiba T, Murata S (2000). T-cell-mediated regulation of osteoclastogenesis by signalling cross-talk between RANKL and IFN-gamma.. Nature.

[29] Zheng H, Yu X, Collin-Osdoby P, Osdoby P (2006). RANKL stimulates inducible nitric-oxide synthase expression and nitric oxide production in developing osteoclasts. An autocrine negative feedback mechanism triggered by RANKL-induced interferon-beta via NF-kappaB that restrains osteoclastogenesis and bone resorption.. J Biol Chem.

[30] Choi SJ, Reddy SV, Devlin RD, Menaa C, Chung H (1999). Identification of human asparaginyl endopeptidase (legumain) as an inhibitor of osteoclast formation and bone resorption.. J Biol Chem.

[31] Reddy SV, Kurihara N, Menaa C, Roodman GD (2001). Paget's disease of bone: a disease of the osteoclast.. Rev Endocr Metab Disord.

[32] Wise GE, Fan W (1989). Changes in the tartrate-resistant acid phosphatase cell population in dental follicles and bony crypts of rat molars during tooth eruption.. J Dent Res.

[33] Yao S, Pan F, Wise GE (2007). Chronological gene expression of parathyroid hormone-related protein (PTHrP) in the stellate reticulum of the rat: implications for tooth eruption.. Arch Oral Biol.

[34] Wise GE, Lin F, Zhao L (1995). Transcription and translation of CSF-1 in the dental follicle.. J Dent Res.

[35] Takayanagi H, Kim S, Koga T, Nishina H, Isshiki M (2002). Induction and activation of the transcription factor NFATc1 (NFAT2) integrate RANKL signaling in terminal differentiation of osteoclasts.. Dev Cell.

[36] Espinosa L, Paret L, Ojeda C, Tourneur Y, Delmas PD (2002). Osteoclast spreading kinetics are correlated with an oscillatory activation of a calcium-dependent potassium current.. J Cell Sci.

[37] Xiao Z, Hao Y, Qin T, Han Z (2003). Periodic oscillation of blood leukocytes, platelets, and hemoglobin in a patient with chronic eosinophilic leukemia.. Leuk Res.

[38] Go RS (2005). Idiopathic cyclic thrombocytopenia.. Blood Rev.

[39] Fogarty PF, Stetler-Stevenson M, Pereira A, Dunbar CE (2005). Large granular lymphocytic proliferation-associated cyclic thrombocytopenia.. Am J Hematol.

[40] Horwitz M, Benson KF, Duan Z, Li FQ, Person RE (2004). Hereditary neutropenia: dogs explain human neutrophil elastase mutations.. Trends Mol Med.

[41] Colijn C, Mackey MC (2005). A mathematical model of hematopoiesis: II. Cyclical neutropenia.. J Theor Biol.

[42] Defranoux NA, Stokes CL, Young DL, Kahn AJ (2005). In silico modeling and simulation of bone biology: a proposal.. J Bone Miner Res.

